# Migration dynamics of juvenile southern bluefin tuna

**DOI:** 10.1038/s41598-018-32949-3

**Published:** 2018-09-28

**Authors:** Toby A. Patterson, J. Paige Eveson, Jason R. Hartog, Karen Evans, Scott Cooper, Matt Lansdell, Alistair J. Hobday, Campbell R. Davies

**Affiliations:** CSIRO Oceans and Atmosphere, GPO Box 1538 Hobart, Tasmania Australia

## Abstract

Large scale migrations are a key component of the life history of many marine species. We quantified the annual migration cycle of juvenile southern bluefin tuna (*Thunnus maccoyii*; SBT) and spatiotemporal variability in this cycle, based on a multi-decadal electronic tagging dataset. Behaviour-switching models allowed for the identification of cohesive areas of residency and classified the temporal sequence of movements within a migration cycle from austral summer foraging grounds in the Great Australian Bight (GAB) to winter foraging grounds in the Indian Ocean and Tasman Sea and back to the GAB. Although specific regions within the Indian Ocean were frequented, individuals did not always return to the same area in consecutive years. Outward migrations from the GAB were typically longer than return migrations back to the GAB. The timing of individual arrivals to the GAB, which may be driven by seasonality in prey availability, was more cohesive than the timing of departures from the GAB, which may be subject to the physiological condition of SBT. A valuable fishery for SBT operates in the GAB, as do a number of scientific research programs designed to monitor SBT for management purposes; thus, understanding SBT migration to and from the area is of high importance to a number of stakeholders.

## Introduction

Annual migrations allow animals to exploit spatially varying resources such as food or breeding habitat^1,2^. Indeed, migration has been defined as “an adaptive response to spatiotemporal variation in resources that requires individuals to detect and respond to long-range and noisy environmental gradients”^3^. In highly migratory pelagic species, such as bluefin tuna (*Thunnus* spp.), it is unclear why large-scale migrations, which must constitute a significant investment of energy, confer a benefit. While telemetry technology has made migration cycles more generally observable^4–6^, quantifying variability and plasticity of migration schedules remains a challenge^7^. Consequently, some studies of tuna abundance have inferred population declines^8^ and range contractions^9^ without providing context based on an understanding of seasonal availability^10,11^. Sufficient observations now exist for detailed characterization of the seasonal migration dynamics of bluefin tunas, which are some of the world’s most valuable fishery species.

Southern bluefin tuna (*T*. *maccoyii*, SBT, Castelnau 1872), has a wide distribution throughout southern hemisphere waters^12^. The species is thought to comprise a single stock^13^, with several ontogenetic habitat shifts during growth. Juvenile (ages 1–4) SBT aggregate in southern Australian waters throughout the austral summer from south of Western Australia across to eastern parts of the Great Australian Bight (GAB)^14^. From here they disperse westwards into the Indian Ocean or eastwards into the Tasman Sea during the autumn, before returning to the GAB in the following summer^15^. They continue to undertake these seasonal migrations until around age 5 after which they disperse throughout waters in the Pacific, Indian and Atlantic Oceans^14^. Marine predator migrations, particularly for temperate species living in seasonal environments, have been associated with seasonal shifts in the abundance of lower trophic level prey species and juvenile SBT migrations are presumed to represent a strategy for maximizing growth^16^.

Pelagic ecosystems are less easily observed and also exhibit more subtle seasonality than many terrestrial systems. Consequently, determining clear seasonal signals in marine habitats which might drive migratory movements is often difficult.

The juvenile SBT migration cycle has been inferred from seasonal distributions of catch records and from previous archival tagging studies^5,14,17^. Although the migratory dynamics of SBT over a number of years have been generalized by pooling data from many individuals across a number of years^15^, the degree of synchronicity in departure from and return to the GAB and understanding of the environmental drivers associated with migrations to and from the GAB is limited. Here we analyzed a multi-decadal electronic tagging data set from juvenile SBT to: characterize movement rates conditional on behavioural state (resident or migrating); quantify the extent and synchronicity of the migrations of individuals to and from the GAB; and determine key areas of residency and, by association, potentially important habitat. The GAB supports a valuable large-scale fishery for SBT, as well as a number of scientific research programs designed to monitor SBT for international management purposes (Hillary *et al*.^18^). Thus, understanding SBT migration to and from the area is of high importance to an international fishery, and more broadly, it contributes to a better understanding of the entire region, which is one of Australia’s most valuable marine ecosystems^19^.

## Results

### Behavioural classification of movement

**Figure 1:**
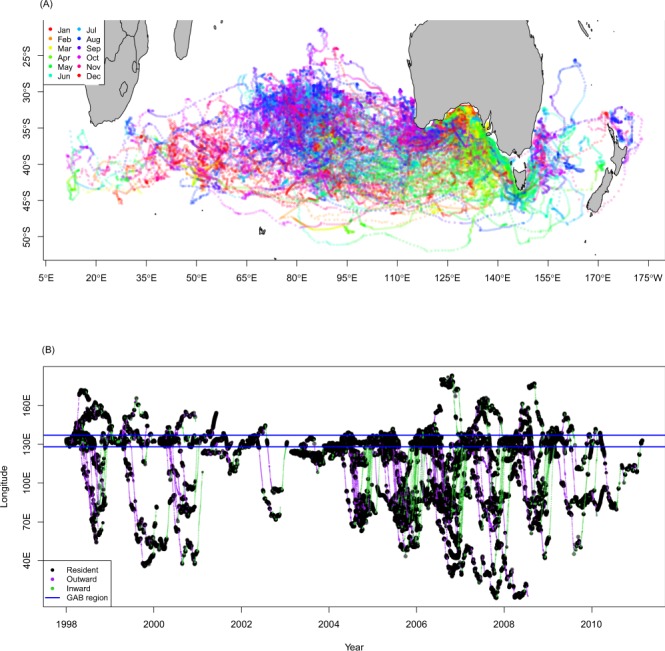
(**A**) Estimated movements of juvenile southern bluefin tuna (coloured by month) derived from deployments of archival tags 1998–2011. (**B**) East-west pattern of migrations of all juvenile southern bluefin tuna coloured according to the most likely movement state classification, with black identifying periods of residence, purple identifying migrations away from the Great Australian Bight (GAB) and green identifying migrations toward the GAB.

The movements from 110 individual juvenile SBT were estimated spanning the period 1998–2011. Cyclic migrations were a defining feature of all estimated archival tag tracks (Fig. 1) with a total of 44 full cycles describing movements outwards and back to the GAB recorded from tags at liberty for a year or more. Only one individual migrated westwards into the Indian Ocean (IO) and remained there for the subsequent summer period rather than returning to the GAB. Juvenile SBT ventured large distances from the GAB (mean 4,262 km, SD 2,181 km, maximum distance 10,251 km) in both westerly and easterly directions (Fig. 1A). The duration of each migration from and back to the GAB varied substantially between individual SBT and ranged from 61–481 days (mean 172 days, SD 89 days). Hidden Markov models (HMMs) showed cyclical migrations in all years of the data set (Fig. 1B).

**Figure 2:**
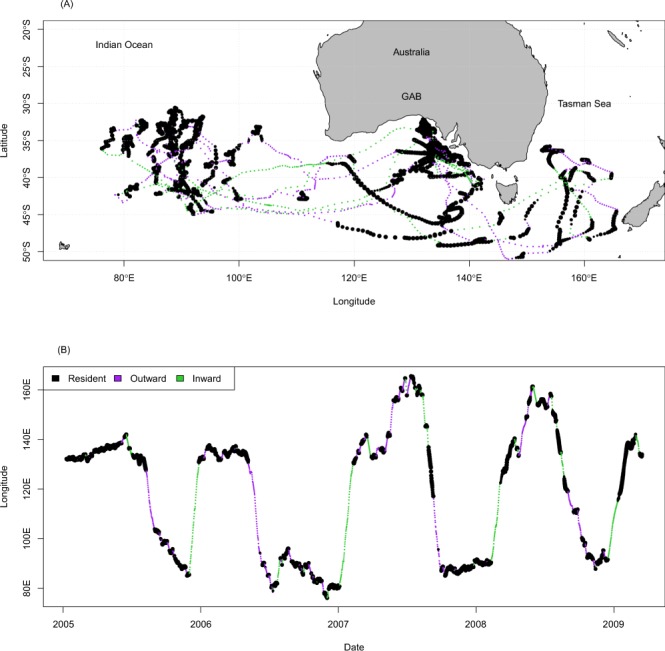
(**A**) The estimated migratory movements of an individual juvenile southern bluefin tuna. Locations are coloured according to the most likely movement state classification, with black identifying periods of residence, purple identifying migrations away from the Great Australian Bight (GAB) and green identifying migrations toward the GAB. (**B**) The migration pattern shown via the predominant east-west (longitude) movement component.

Individuals demonstrated considerable variability in migratory patterns between years (Fig. 1B). For example, one SBT tagged at age 2 made four return migrations between the GAB and the IO across the period 2006–2010, travelling as far west as approximately 80°E (Fig. 2). Its migrations away from the GAB during the first two years were rapid and directed, punctuated by periods of residency before similarly rapid migrations eastwards were initiated. After the first two years of tag deployment, the behaviour of this individual changed: less time was spent in the GAB during the summer period across 2007/08 and 2008/09. Similar patterns were also observed in other long term deployments (Fig. 1B) and indicate that, as juvenile SBT age, they progressively spend less time in the shelf waters of the GAB. Also, in its third and fourth years after tagging, rather than departing the GAB and heading west into the IO as per the previous two years, this SBT moved east and into the Tasman Sea for a period before then heading west into the IO (Fig. 2). This westward migration was also punctuated by a higher occurrence of periods of residence than in the first two years of the deployment. Although this particular fish migrated further west in its second year after tagging than the next two, individuals typically showed greater maximum westward movement in later (older) years of multi-year tag deployments relative to earlier (younger) periods, with the average western-most longitude visited increasing almost linearly with age from 122.7°E at age 1 to 54.4°E at age 6. This trend of increased westward and southward extent of migrations with age was apparent throughout the data-set (see Fig. S5, Table S3). Overall the SBT shown in Fig. 2 is estimated to have travelled 111,883 km in the 4.18 years observed.

**Figure 3:**
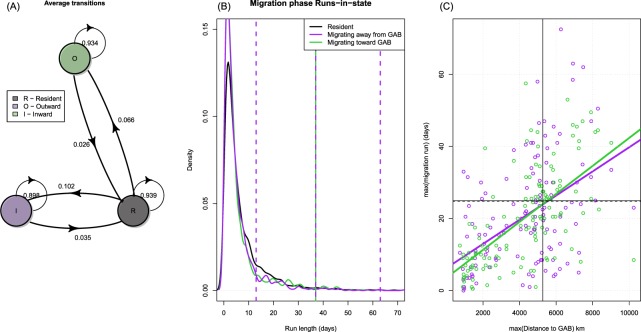
(**A**) Structure of the assumed Markov model chain for transitions between behavioural modes. Probabilities of transitions (arrows) label the transition between modes (circles). (**B**) Run lengths (lengths of runs in a single state) within behavioural modes when southern bluefin tuna were outside the Great Australian Bight (GAB). (**C**) Relationship between the time-in-migration mode and maximum distance from the GAB.

**Figure 4:**
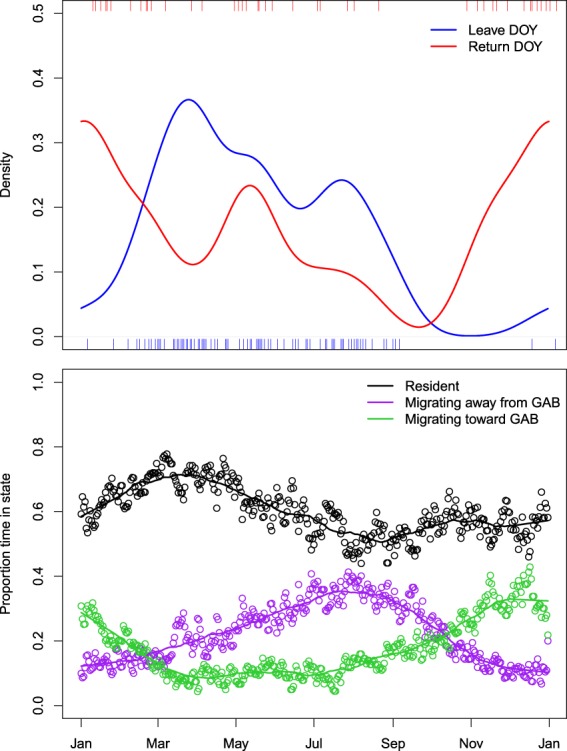
(**A**) Circular densities of juvenile southern bluefin tuna migrating away from the Great Australian Bight (GAB) (blue) and toward the GAB (red) by day of the year (DOY). The rug plots show calculated departure date (bottom x-axis) and return (top x-axis). Shown also (vertical lines) are the peak periods of return (DOY = 0) and departure (DOY = 147). (**B**) Proportion of positions in each behaviour mode by day-of-year.

HMMs classified SBT as being in a resident mode for nearly 60% of the time observed, with outward migrations from the GAB containing more periods of residence than inward migrations to the GAB (Table 1). There was a high persistence within a state (Fig. 3) with >90% probability of remaining in the current state between two consecutive steps. Run lengths within states showed similar distributions – typically <10 movement steps - although the resident mode had a predominance of longer run lengths (Fig. 3). Although quite variable, the time individuals were classified as migrating demonstrated a positive relationship with the maximum distance to the GAB (Fig. 3), suggesting that the further individual SBT roam from the GAB, the more time they spend migrating. On leaving the GAB, most juvenile SBT (84%) moved westwards into the IO rather than eastwards towards the Tasman Sea. Average distances within movement mode were estimated to be 25% higher during movements away from the GAB compared to return movements (Table 1), but with high variance relative to mean movement distance. Residency in the GAB was generally confined to the first 150 days of the calendar year with departure dates of individuals being highly variable. Departures generally began in February with overall residency in the GAB starting to decline by the end of March (Fig. 4).

**Table 1:** The proportion of time spent in each behavioural state, associated interquartile range (IQR) and state dependent distribution parameters (mean and standard deviation (SD)) estimated by the hidden Markov model based on all estimated tracks from tagged juvenile southern bluefin tuna.

Behavioural state	Proportion	IQR	Mean	SD
Resident	0.57	0.19	0.00	14.00
Outward	0.25	0.14	39.02	21.58
Inward	0.18	0.11	31.08	22.65

The numbers of juvenile SBT returning to the GAB increased from November, peaking in December-January and continuing through to March (Fig. 4), reflecting the extended period of residency associated with the GAB. There was evidence of a secondary peak in return times to the GAB in May but this may be due to increased variability through reduced sample sizes (N = 44 return dates). The departure of juvenile SBT from the GAB began in late February and early March and trailed off into September (Fig. 4). There was some evidence of a secondary peak in departures in July. Although there were more observations of departures, as the large majority of tags were released in the GAB (N = 131), the sample sizes were relatively small and further data is required to determine whether there is finer structure in GAB departure and arrival dates. There was little evidence of a relationship with age and the timing of departure and arrival in the GAB (Supplementary Fig. S6).

### Areas of residence and relationships with habitat descriptors

**Figure 5:**
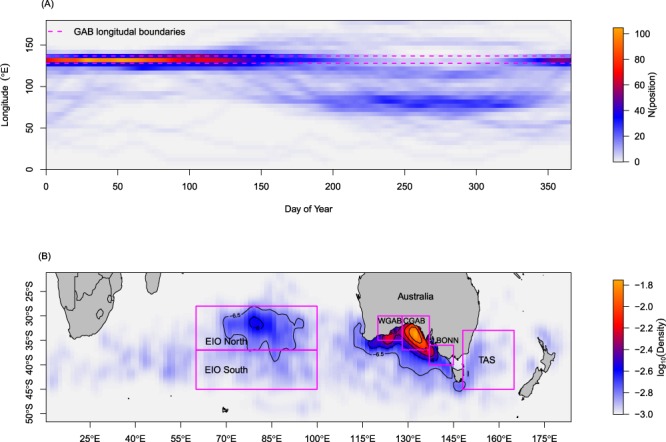
(**A**) Frequency of longitudes (colour) by day-of-year (DOY). (**B**) Kernel density estimates of locations classified as resident, identifying six main residency regions north-eastern Indian Ocean (EIO North), south-eastern Indian Ocean (EIO South), western Great Australian Bight (WGAB), central Great Australian Bight (CGAB), the Bonney coastline (BONN) and the Tasman Sea (TAS).

Examining only the east-west components of movement with respect to time of year shows the clear residency period in the GAB followed by a propensity to move to longitudes between 70–100°E (Fig. 5A). Kernel density estimation refined this general pattern and identified three areas associated with residency of juvenile SBT in the eastern IO (north EIO), western GAB and central GAB. For comparison we also considered habitat in adjacent areas of the southern EIO, the upwelling area along the Bonney coastline in western Victoria, which is an area of locally high productivity, and the Tasman Sea (Fig. 5). Juvenile SBT in our dataset were absent from the Tasman Sea in summer despite this being, historically, an area of high summer catches of SBT via a surface purse seine fishery^12^ and also an important winter residence area for sub-adult and adult SBT^20,21^.

Productivity in all residency regions demonstrated a clear seasonal cycle, with productivity in the north and south EIO areas tending to be reduced relative to other residency areas (Supplementary Fig. S4). The GAB consistently had higher surface chlorophyll maximums and a stronger seasonal cycle in comparison to the other residency areas, with maxima occurring during the austral winter. Chlorophyll maximums in the GAB were also out of phase with the three most distant residency areas, with maxima in the north EIO, south EIO and Tasman Sea tending to occur across the austral spring and summer months. Of all areas, the magnitude of the seasonal signal in surface productivity was lowest in the north EIO.

**Figure 6:**
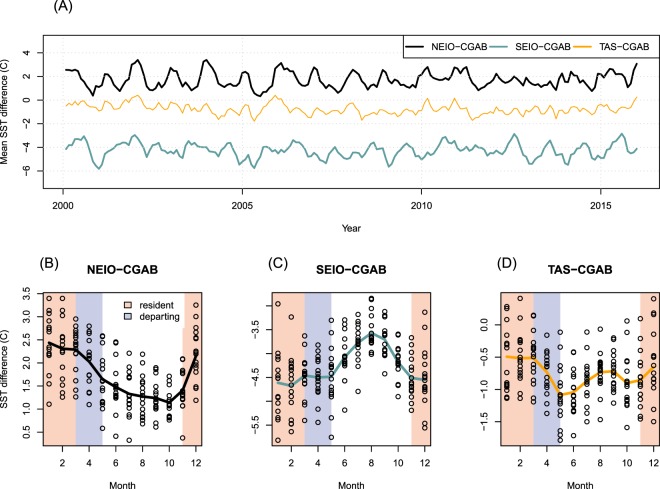
(**A**) Time series of the difference in sea surface temperature (SST) values from the central Great Australian Bight (CGAB) and those in the north-eastern Indian Ocean (NEIO), south-eastern Indian Ocean (SEIO) and Tasman Sea (TAS). (**B**) Monthly SST differences between the CGAB and the NEIO, SEIO and TAS areas across all years pooled. Lines highlight trends. Note that the y-axis scales vary between plots.

Mean sea surface temperatures (SST) were more temporally consistent between regions (Fig. 6). If we consider the central GAB (CGAB) as the “core” summer region for juvenile SBT, and compare SST from the CGAB with the EIO and Tasman Sea, the south EIO was on average (across all years and months) 4.3 °C cooler (SD = 0.61 °C), the north EIO was on average 1.7 °C warmer (SD = 0.67 °C), and the Tasman Sea was on average 0.75 °C cooler (SD = 0.43 °C) than the CGAB (Fig. 6). While the SBT tagged in this study are all juvenile, there were substantial changes in size over the age classes contained in this data (ages 1–6). The spatial distribution changed accordingly with SBT progressively migrating further west and into cooler water as they aged (Supplementary Materials Fig. S5, Table S3).

In contrast to younger (age 1) SBT, which remained year round in the highest productivity shelf waters^22^ within and to the west of the GAB (Supplementary Materials Fig. S5), older fish showed more complex patterns with regard to productivity. Juvenile SBT aged 2+ were resident in the GAB during periods of lower productivity and higher SST when compared to productivity and SST in other areas, and absent from the GAB during the period when the GAB’s highest surface measurements of chlorophyll were observed (Figs 6 and S4). During their migrations to the IO, juvenile SBT encountered a range of SST conditions that varied predictably with latitude. Periods of residence in the IO were characterized by lower surface productivity and cooler temperatures than those of the GAB at the same time. Individuals migrating into the Tasman Sea appeared to experience relatively consistent SST and chlorophyll surface conditions when compared with other areas.

## Discussion

The migration and distribution patterns presented here show that, in common with other large animals utilizing temperate latitudes, juvenile SBT undertake long distance migrations. Where SBT, like other bluefin, are perhaps atypical, is that these zonal cyclic migrations are specific to juveniles, and there is no obvious latitudinal (meridional) component that could be associated with seasonal temperature. Nor did we find evidence of correlation with environmental drivers such as SST and surface chlorophyll that have been found to be important in migration for other species. While many juvenile SBT were observed to consistently migrate to key areas, such as the central IO during winter, individuals were observed to vary where they spent their winter period from year to year and even within a year. A number of individuals were observed to spend one winter in the IO and the next in the Tasman Sea or even displaying periods of residence in both locations within the same winter period. Similarly, the departure and arrival dates of juvenile SBT to and from the GAB differed markedly between individuals. Yet despite this variability between individuals, juvenile SBT consistently return to the GAB over the age-classes tagged, even if they spend subsequent winters in different oceanic regions. In addition, their summer spatial usage within the GAB is relatively stable across the two decades for which electronic tagging data has been collected. There was evidence of reduced usage of the GAB during summer in the age 5+ SBT, which is consistent with the notion of the thermal niche of SBT with growth.

The estimated differences in speed of inward and outward movement (Table 1) may result from a higher searching time (and therefore a need to cover greater distances) required when locating inter-annually variable winter foraging grounds. Inward migrations to the GAB, being a return to a “known” and geographically confined region, may require less search effort and, in association, the need to cover lower distances in the annual movement cycle.

Unlike many other migrating species, such as^23,24^ that demonstrate remarkably synchronized timing of movements, the dates at which individual SBT departed and returned to the GAB were highly variable. This suggests that the cues for juvenile SBT to depart from, or return to, the GAB may be related to environmental variability rather than fixed cues such as day-length. For a fast growing, immature but, ultimately large, ocean predator, such as juvenile SBT, identifying abundant food sources and maximizing growth is likely to be the key driver of their behaviour. If movements are related to prey availability, then movements may be triggered by declines in prey at the current location, or anticipation of higher prey levels at the destination.

The duration or spatial extent of residency did not directly coincide with seasonal cycles in surface primary productivity. Areas where SBT were predominantly in resident mode were consistently associated with low surface productivity, a finding that is consistent with previous studies on SBT^17^, but inconsistent with studies on other bluefin tunas where movements were found to track seasonal productivity maxima^25,26^. Several explanations are possible. First, it is possible, that juvenile SBT, being visual predators, prefer to hunt their prey in clear waters away from areas of high turbidity associated with high levels of primary productivity^27–29^. Second, the energy transfer from primary to intermediate levels in the food web of pelagic predators, such as SBT, involves a time lag. As intermediate trophic species increase in abundance, resources of primary species are consumed and decline in abundance and, as a result, the presence of higher order predators may be offset from high levels of primary productivity^30,31^. Third, areas of concentrated productivity are likely to operate at smaller spatial scales than those at the scale that residency associations were investigated. Eastern parts of the GAB and the area of the Bonney coastline east of the GAB are areas of seasonal coastal upwelling driven by south-easterly winds primarily during the austral summer and autumn, leading to enhanced phytoplankton biomass^32^. These upwelling features tend to be spatially limited and may only operate across short temporal scales of only a few days^33,34^. Finally, satellite-derived indices of productivity only provide an indication of productivity in the surface waters. Productivity maxima in the GAB are often at depth^34^, suggesting that total productivity may not be highly correlated with satellite measurements. In other regions, the distributions of pelagic predators such as tuna have been associated with physical features of the ocean such as bathymetry, frontal features and subsurface structure of the water column^28,35^. It may be more appropriate to investigate the interaction between this sort of physical features and sub-surface water-masses, rather than indicators of surface primary productivity as potential drivers of large-scale migration patterns.

A number of seasonal populations of small pelagic fish have been observed to occur in the GAB. A large fishery primarily targeting Australian sardine or pilchard (*Sardinops sagax*) occurs in the region throughout the austral summer and early autumn months when the species is spawning^36–38^. This coincides with when juvenile SBT are in the GAB and populations of these species almost certainly provide an important seasonal prey resource for SBT^37,38^. Consumption of spawning, energy-rich small pelagic fish may result in faster growth for juvenile SBT than might be attained by foraging on equivalent or greater biomass of alternative prey sources elsewhere. Once these energy-rich prey conclude spawning, migration to areas outside the GAB may be favored. Studies on Pacific and Atlantic bluefin tuna support this hypothesis^39,40^, suggesting specialization on high calorie forage species such as sardine, herring and anchovy when abundant. However, little is known of the diet of juvenile SBT outside of the GAB, with the small amount of information available suggesting that their diet is more varied in areas outside the GAB^41^. A better understanding of the intermediate trophic links and the efficiency of trophic transfer from primary production into SBT prey species, as well as measures of prey ingestion and body condition during their residence within the GAB, might provide greater insight into the drivers of residence and migration behaviours.

Thermal regimes could also be important for growth and may provide additional drivers for the seasonal utilization of the GAB by juvenile SBT. Growth studies of juvenile SBT have shown that a large proportion of annual growth occurs over the months when fish are likely in the GAB^42^. This could indicate that higher temperatures infer physiological benefits, via enhanced digestive throughput^43^, without imposing metabolic costs. Quantitative assessments of the influence of water temperature on growth rates and digestion in tunas are largely lacking^44,45^, with most understanding of the influence of temperature on growth based on modelling approaches.

Patterns of historical fisheries exploitation of the species may also play a role in determining residence areas observed. Reduction in the average recruitment associated with overfishing and the depleted state of the stock^46^ has likely impacted the distribution of juvenile SBT. Data from the scientific aerial survey used to monitor recruitment to the stock since the early 1990s^47^ revealed juveniles are largely confined to the shelf waters and away from the western GAB. This distribution was particularly evident during a period of historically low recruitments from the late 1990s through to around 2007. In the years after 2007, the distribution started to expand, with the distribution in 2010 and 2013–2016 being similar to the period of higher average recruitment prior to the late 1990s. Recent years have seen indications of relatively strong recruitment^48^. Should this trend continue, the associated increase in the abundance of juvenile SBT may result in changes to the distribution of this component of the population.

## Conclusions

This paper has described the characteristics of the annual migration of juvenile SBT, showing alternating patterns of residence and migratory behavior. Our results provide important insights into the variability of migration behaviours between individuals within the same time period and within individuals across multiple years. The proximate drivers for these long-distance migrations remains unclear. Evolutionary arguments would suggest that SBT migrations are an evolved response to seasonal changes in resources. The complexity of migration routes, their timing and apparent mismatch to indices of ocean temperature and surface productivity suggests that greater understanding of the diet of juvenile SBT, the associated distribution of prey fields and their relationships to not only the marine environment, but the physiology of individuals, is required. Only with this understanding will the drivers of such a large scale migration and its fitness gains when compared to adopting a more resident strategy be revealed.

## Methods

### Tag deployment and geolocation

Archival tags (Wildlife Computers, Redmond, USA) were deployed in juvenile SBT of lengths 48–150 cm as part of a series of tagging programs during the 1990s and 2000s^15,17^. All tagging was carried out in accordance with the CSIRO Code of Practice for tagging marine animals, with protocols used approved by the Tasmanian Government Department of Primary Industries, Parks, Water and the Environment Animal Ethics committee. Electronic tags consisted of 229 Mk7 and 597 Mk9 archival tags deployed occurred across five main areas spanning the range of juveniles: South Africa (n = 27), the mid-Indian Ocean (n = 159), West Australia (n = 204), the GAB (n = 351) and New Zealand (n = 85). Recapture of tags was facilitated through the international commercial fisheries fleet catching SBT, with 19% returned at the time of analysis.

Geolocation, the process of estimating position from light data collected by each archival tag, was performed using a state-space modelling approach detailed in^49^ and following the general methodology detailed in^50,51^. The likelihood surfaces generated were then input to a grid-based HMM incorporating sea surface temperature and maximum depth to estimate a “most probable track” for each animal (see Supplementary Material). The outputs from the HMM were treated as locations for input to the behaviour switching models described next.

### Hidden Markov models of migration

Hidden Markov Models (HMMs)^52,53^ were used to estimate when individuals were migrating (directed fast movement covering large distances) and when individuals were resident (undirected slow movement with localized movements).

The longitudes estimated by the geolocation process and associated estimated tracks indicated that juvenile SBT undertake primarily east-west migrations within a relatively limited latitudinal range. The model used distance from a central location (32°S, 130°E) in the GAB (*d*_*GAB*_) as a point of reference to determine whether SBT were resident (with no directed movement away from or toward the GAB), migrating outwards (i.e. demonstrating consistent increasing *d*_*GAB*_) or undertaking a return migration (i.e. consistent decreasing *d*_*GAB*_).

We constructed three-state HMMs, with latent states labelled (1) “resident”, (2) “outward migration” and (3) “inward migration”. We assume that the probabilities of switching from one state to another are constant over time, so that ${{\bf{x}}}_{t}={\bf{P}}{{\bf{x}}}_{t-1}\,$where the transition matrix **P** is defined as

$${\bf{P}}=(\begin{array}{ccc}{\pi }_{1,1} & {\pi }_{1,2} & {\pi }_{1,3}\\ {\pi }_{2,1} & {\pi }_{2,2} & 0\\ {\pi }_{3,1} & 0 & {\pi }_{3,3}\end{array})$$

Here *π*_*i*,*j*_ is the probability of moving from state *i* to state *j*. This transition matrix implies that individuals can go from the resident state to either of the migratory states, or remain resident. When in a migratory state, individuals can only remain in that state or transition to resident. Transitions between the two migratory states can only be via the resident state, but note this can occur after a single-time-step. Because the values in each row must sum to one, e.g., ${\pi }_{1,3}=1-{\pi }_{1,1}-{\pi }_{1,2}$, this means only four parameters in the transition matrix need to be estimated.

For those individuals that only made a migration in one direction (primarily because individuals were caught or their tag failed prior to return to the GAB), a two-state model (with resident and migration states only) was used. In this case, the transition matrix was a 2 × 2 matrix containing only the first two rows and columns of **P** above.

For the observation model, the likelihood function *g*(.) is given by a normal distribution with state-specific mean *μ*_*i*_ and variance *σ*^2^, for example for state *i* and distance moved *d*_*t*_: ${g}_{i}({\mu }_{i},{\sigma }_{i},{d}_{t})={(\sqrt{2\pi {\sigma }_{i}})}^{-1}$ $\,\exp \,(\,-\,{({d}_{t}-{\mu }_{i})}^{2}/2{\sigma }^{2}$. Note transition probabilities were assumed constant over time.

### Categorization and examination of migratory behaviour

Categorization of movements into the three states allowed for the calculation of: (i) time spent in each state; (ii) the run length of each state (time spent consecutively in each behavioural state); (iii) the proportion of the tagged fish in each behavioural state as a function of day of year; (iv) timing of the initiation of migration away from the GAB (as day of the year); (v) timing of the return of each individual to the GAB (as day of the year). The spatial distribution of behaviours was also mapped along the estimated track of each individual providing for an aggregate picture of intra-annual migration behaviours. Non parametric smoothing splines were used to detect annual trends^54^.

Because individual tracks of SBT can comprise resident periods interspersed with periods of migration, tracks were divided into “trips” based on a threshold of moving 500 km from the central GAB locus (chosen at 32°S, 130°E, as described above). Position estimates within this radius were classified as being within the GAB. We adopted the criteria that the beginning of a consecutive run of 120 positions where *d*_*GAB*_ > 500 km was the departure date and the return was the date at which *d*_*GAB*_ < 500 km. Empirical circular density distributions^55^ were used to examine annual timing or departure and return of SBT to the GAB.

### Association between areas of residence and environmental parameters

We applied kernel density estimation^56^ to the mode of locations classified as resident to create a map of the core residence locations of SBT throughout their range. These were then used to guide extractions of remotely sensed values of SST and chlorophyll-a, as an indicator of productivity (see Supplementary Material). Once extracted, the characteristics of the ocean environment (based on SST and chlorophyll-a) were compared between areas of residence, and their relationship with residency behaviour in SBT examined.

## Electronic supplementary material

Supplementary material

## References

[1] Alerstam T, Hedenström A, Åkesson S (2003). Long‐distance migration: evolution and determinants. Oikos.

[2] Durant JM (2005). Timing and abundance as key mechanisms affecting trophic interactions in variable environments. Ecol. Lett..

[3] Guttal V, Couzin ID (2010). Social interactions, information use, and the evolution of collective migration. Proc. Natl. Acad. Sci..

[4] Costa DP (1993). The secret life of marine mammals: Novel tools for studying their behaviour and biology at sea. Oceanography.

[5] Gunn J, Block B (2001). Advances in acoustic, archival, and satellite tagging of tunas. Fish Physiol..

[6] Block B (2011). Tracking apex marine predator movements in a dynamic ocean. Nature.

[7] Young JW (2015). The trophodynamics of marine top predators: Current knowledge, recent advances and challenges. Deep Sea Res. Part II Top. Stud. Oceanogr..

[8] Worm B, Sandow M, Oschlies A, Lotze HK, Myers RA (2005). Global patterns of predator diversity in the open oceans. Science.

[9] Worm B, Tittensor DP (2011). Range contraction in large pelagic predators. Proc. Natl. Acad. Sci..

[10] Lutcavage ME (2000). Tracking adult North Atlantic bluefin tuna (Thunnus thynnus) in the northwestern Atlantic using ultrasonic telemetry. Mar. Biol..

[11] Block BA (2005). Electronic tagging and population structure of Atlantic bluefin tuna. Nature.

[12] Caton, A. E. Review of aspects of southern bluefin tuna biology, population, and fisheries. *FAO Fish*. *Tech*. *Pap*. *FAO* (1994).

[13] Grewe P, Elliott N, Innes B, Ward R (1997). Genetic population structure of southern bluefin tuna (Thunnus maccoyii). Mar. Biol..

[14] Hobday, A. J. *et al*. Distribution and Migration—Southern Bluefin Tuna (*Thunnus maccoyii*). *Biol*. *Ecol*. *Bluefin Tuna* 189 (2015).

[15] Basson, M., Hobday, A., Eveson, JP & Patterson, TA. *Spatial interactions among juvenile southern bluefin tuna at the global scale: A large scale archival tag experiment*. (Report to the Fisheries Research and Development Corporation, 2012).

[16] Roff DA (1983). An allocation model of growth and reproduction in fish. Can. J. Fish. Aquat. Sci..

[17] Bestley S, Patterson TA, Hindell MA, Gunn JS (2010). Predicting feeding success in a migratory predator: integrating telemetry, environment, and modeling techniques. Ecology.

[18] Hillary Richard M, Preece Ann L, Davies Campbell R, Kurota Hiroyuki, Sakai Osamu, Itoh Tomoyuki, Parma Ana M, Butterworth Doug S, Ianelli James, Branch Trevor A (2015). A scientific alternative to moratoria for rebuilding depleted international tuna stocks. Fish and Fisheries.

[19] Baghurst B (2017). Findings from the Great Australian Bight Research Program–an integrated study of environmental, economic and social values. APPEA J..

[20] PATTERSON TOBY A., EVANS KAREN, CARTER THOR I., GUNN JOHN S. (2008). Movement and behaviour of large southern bluefin tuna (Thunnus maccoyii) in the Australian region determined using pop-up satellite archival tags. Fisheries Oceanography.

[21] Evans K, Patterson TA, Reid H, Harley SJ (2012). Reproductive schedules in southern bluefin tuna: are current assumptions appropriate?. PloS One.

[22] FUJIOKA KO, HOBDAY ALISTAIR J., KAWABE RYO, MIYASHITA KAZUSHI, HONDA KENTARO, ITOH TOMOYUKI, TAKAO YOSHIMI (2010). Interannual variation in summer habitat utilization by juvenile southern bluefin tuna (Thunnus maccoyii) in southern Western Australia. Fisheries Oceanography.

[23] Bauer S, Lisovski S, Hahn S (2016). Timing is crucial for consequences of migratory connectivity. Oikos.

[24] Gunnarsson T, Gill J, Sigurbjörnsson T, Sutherland W (2004). Pair bonds: arrival synchrony in migratory birds. Nature.

[25] Boustany AM, Matteson R, Castleton M, Farwell C, Block BA (2010). Movements of pacific bluefin tuna (Thunnus orientalis) in the Eastern North Pacific revealed with archival tags. Prog. Oceanogr..

[26] Whitlock RE (2015). Direct quantification of energy intake in an apex marine predator suggests physiology is a key driver of migrations. Sci. Adv..

[27] Laurs RM, Fiedler PC, Montgomery DR (1984). Albacore tuna catch distributions relative to environmental features observed from satellites. Deep Sea Res. Part Oceanogr. Res. Pap..

[28] Zagaglia CR, Lorenzzetti JA, Stech JL (2004). Remote sensing data and longline catches of yellowfin tuna (Thunnus albacares) in the equatorial Atlantic. Remote Sens. Environ..

[29] White WB, Gloersen KA, Marsac F, Tourre YM (2004). Influence of coupled Rossby waves on primary productivity and tuna abundance in the Indian Ocean. J. Oceanogr..

[30] Lehodey, P. Climate and fisheries: an insight from the central Pacific Ocean. *Mar*. *Ecosyst*. *Clim*. *Var*. *N*. *Atl*. *Oxf*. *Univ*. *Press Oxf*. 137–146 (2004).

[31] Hazen EL (2013). Scales and mechanisms of marine hotspot formation. Mar. Ecol. Prog. Ser..

[32] McClatchie, S., Middleton, J. F. & Ward, T. M. Water mass analysis and alongshore variation in upwelling intensity in the eastern Great Australian Bight. *J*. *Geophys*. *Res*. *Oceans* **111** (2006).

[33] Schahinger RB (1987). Structure of coastal upwelling events observed off the south-east coast of South Australia during February 1983–April 1984. Mar. Freshw. Res..

[34] Middleton JF, Bye JA (2007). A review of the shelf-slope circulation along Australia’s southern shelves: Cape Leeuwin to Portland. Prog. Oceanogr..

[35] Teo SL, Boustany AM, Block BA (2007). Oceanographic preferences of Atlantic bluefin tuna, Thunnus thynnus, on their Gulf of Mexico breeding grounds. Mar. Biol..

[36] Kemps, H., Totterdell, J., Gill, H. & Nishida, T. Preliminary analysis on the diet and feeding ecology of juvenile southern bluefin tuna, Thunnus maccoyii, in relation to the southern coastal waters of Western Australia. In ‘Report to the tenth workshop, SBT recruitment and monitoring program’ (1998).

[37] Ward TM (2006). Pelagic ecology of a northern boundary current system: effects of upwelling on the production and distribution of sardine (Sardinops sagax), anchovy (Engraulis australis) and southern bluefin tuna (Thunnus maccoyii) in the Great Australian Bight. Fish. Oceanogr..

[38] Itoh T, Kemps H, Totterdell J (2011). Diet of young southern bluefin tuna Thunnus maccoyii in the southwestern coastal waters of Australia in summer. Fish. Sci..

[39] Madigan DJ, Baumann Z, Fisher NS (2012). Pacific bluefin tuna transport Fukushima-derived radionuclides from Japan to California. Proc. Natl. Acad. Sci..

[40] Golet WJ (2015). The paradox of the pelagics: why bluefin tuna can go hungry in a sea of plenty. Mar. Ecol. Prog. Ser..

[41] Serventy D (1956). The Southern Bluefin Tuna, Thunnus thynnus maccoyii (Castelnau), in Australian Waters. Mar. Freshw. Res..

[42] Eveson JP, Laslett GM, Polacheck T (2004). An integrated model for growth incorporating tag recapture, length frequency, and direct aging data. Can. J. Fish. Aquat. Sci..

[43] Stevens E, McLeese J (1984). Why bluefin tuna have warm tummies: temperature effect on trypsin and chymotrypsin. Am. J. Physiol.-Regul. Integr. Comp. Physiol..

[44] Clark T. D., Brandt W. T., Nogueira J., Rodriguez L. E., Price M., Farwell C. J., Block B. A. (2010). Postprandial metabolism of Pacific bluefin tuna (Thunnus orientalis). Journal of Experimental Biology.

[45] Chapman EW, Jørgensen C, Lutcavage ME (2011). Atlantic bluefin tuna (Thunnus thynnus): a state-dependent energy allocation model for growth, maturation, and reproductive investment. Can. J. Fish. Aquat. Sci..

[46] Polacheck T (2012). Assessment of IUU fishing for southern bluefin tuna. Mar. Policy.

[47] Eveson, P. & Farley, J. *The aerial survey index of abundance: 2016 updated results*. (CCSBT-ESC/1609/09, 2016).

[48] Commission for the Conservation of Southern Bluefin Tuna. *Report of the twenty first meeting of the Scientific Committee*, *10 September*, *Kaohsiung*, *Taiwan* (2016).

[49] Basson Marinelle, Bravington Mark V., Hartog Jason R., Patterson Toby A. (2016). Experimentally derived likelihoods for light-based geolocation. Methods in Ecology and Evolution.

[50] Pedersen MWever, Patterson TA, Thygesen UH, Madsen H (2011). Estimating animal behavior and residency from movement data. Oikos.

[51] Thygesen, U. H., Pedersen, M. W. ever & Madsen, H. Geolocating fish using hidden Markov models and data storage tags. in *Tagging and Tracking of Marine Animals with Electronic Devices* 277–293 (Springer, 2009).

[52] Patterson TA, Basson M, Bravington MV, Gunn JS (2009). Classifying movement behaviour in relation to environmental conditions using hidden Markov models. J. Anim. Ecol..

[53] Zucchini, W. & MacDonald, I. L. *Hidden Markov models for time series: an introduction using R*. (CRC Press, 2009).

[54] Friedman, J. H. *A variable span smoother*. (DTIC Document, 1984).

[55] Agostinelli, C. & Lund, U. R package circular: Circular Statistics (version 0.4-93). *URL**https://r-forge.r-project.org/projects/circular/* (2017).

[56] Venables W. N., Ripley B. D. (2002). Modern Applied Statistics with S.

