# Supplementary material for: Migration dynamics of juvenile southern bluefin tuna
Source: Sci Rep. 2018 Sep 28;8:14553. doi: 10.1038/s41598-018-32949-3 (PMC6162310; doi:10.1038/s41598-018-32949-3)
Supplement: Supplementary file 1 — Supplementary material [file 41598_2018_32949_MOESM1_ESM.docx]

Supplementary material for: Migration dynamics of juvenile southern bluefin tuna

Toby A. Patterson*, J. Paige Eveson, Jason R. Hartog, Karen Evans, Scott Cooper, Matt Lansdell, Alistair J. Hobday, Campbell R. Davies

CSIRO Oceans and Atmosphere, GPO Box 1538 Hobart, Tasmania, Australia

*Email: Toby.Patterson@csiro.au

1. Calculation of observation error models for state-space model estimation

A discrete space and time state space model was used to estimate the locations of SBT from electronic tag data. Typically state-space models are expressed as a set of coupled models --

1. A process model $g$ describing the movement dynamics of the individual animal:

$$\mathbf{X}_{\mathbf{t}}=g(\mathbf{X}_{t-1})+\eta_{t}$$

2. The observation model which relates observations to the true (hidden) location:

$$\mathbf{Z}_{t}=h(\mathbf{X}_{t})+\varepsilon_{t}$$

Here $\mathbf{X}_{t}$ is an estimate of the hidden state of the system, in this case the location in space $\{lon_{t}, lat_{t}\}$ at time $t$, and $\mathbf{Z}_{t}$ is an associated, noisy observation of the process. The $g(.)$ and $h(.)$ are the process and observation models which respectively give the probability of a hidden location state and the probability of that state given the observations. The terms $\eta$ and $\varepsilon$ are the associated process and observation error distributions.

**1.1 Process model**

The movement /process model used operates on a discrete state space that consists of a grid of points that are assumed to be the midpoint of a grid cell. Let the grid of locations be referred to as: $[x_{1},y_{1},x_{1},y_{2},\ldots,x_{n-1}y_{n},\ldots x_{n},y_{n}]$ . In order to constrain estimated tracks to valid locations (e.g. the ocean), grid cells on land are removed from $\boldsymbol{X}$ *a priori*. The probability distribution of position estimates is described by the state vector $\mathbf{X}_{t}$ and movements between grid locations are governed by a Markovian transition matrix $\boldsymbol{\Gamma}$ whose are given by the half-normal distribution with parameter $\theta$. If a random variable $X\sim N(0,\sigma^{2})$, then $Y=|X|$ follows a half normal distribution given by:

$$f_{Y}(y;\theta)=\frac{2\theta}{\pi}\exp\left( -\frac{y^{2}\theta^{2}}{\pi} \right)$$

where $\theta=\frac{\sqrt{\pi}}{\sigma\sqrt{2}}$. In this case, the values of $y=GCD(p_{1},p_{2})$ where $p_{i}=\{x_{i},y_{i}\}$ is the Great Circle Distance (GCD) between latitude/longitude pairs on the grid. The values from the half-normal PDF are normalized so that $\sum_{i} \gamma_{ik}=1$. In order to drastically speed up the computations, we also enforce a maximum distance constraint by choosing a value $d_{max}$ (here a value of 1,400km was used) and set $\Gamma$ to where $GCD(p_{1},p_{2})>d_{max}$ is zero.

This allows compact storage of $\boldsymbol{\Gamma}$ as a sparse matrix as almost all entries are zero and we only store the row and column indices of the non-zero elements and their associated values. Having constructed this transition matrix, given a state vector $\mathbf{X}_{t}$ we can predict the probability of the next location as:

$$\mathbf{X}_{t+\delta t}=\boldsymbol{\Gamma}(\hat{\theta})^{\delta t}\mathbf{X}_{t}$$

The notation $\boldsymbol{\Gamma}(\hat{\theta})$ is intended to denote that the values in the transition matrix are a function of the estimated $\hat{\theta}$.

**1.2 Error model**

To create error distributions for state-space models, three data sources were combined:

- 1. Light sensor data, used to construct twilight likelihoods^1^;
  2. Sea surface temperature (SST) measured from the tags, compared with remotely sensed SSTs;
  3. Maximum dive depth as measured by the tags, compared with bathymetry information.

We assume that the estimates of light (*L*), SST (*T*) and maximum depth (*D*) obtained from the tags are independent. Therefore the total data likelihood over candidate grids cell in the state space $\mathbf{Z}$ is simply the product of the individual likelihood surfaces over the same state space;

$$[\mathbf{Z}_{t}|\mathbf{X}_{t}]=\mathcal{L}_{SST}(\Delta T_{t})\times\mathcal{L}_{Light}(L_{t})\times\mathcal{L}_{depth}(\Delta D_{t})$$

Given details of twilight likelihood construction are detailed elsewhere^1^, we describe likelihood components 2 and 3 only.

**1.2.1 Sea surface temperatures**

Temperature sensor measurements from a tag, taken at, or near the ocean surface provide a noisy estimate of SST. Calibration of an error model to be incorporated into a state-space model involves comparing observations from tags with remote-sensing data. To do this, we compared measures of SST from 99 drifting popup satellite archival tags (PSATs)[[1]](#footnote-1) (PAT3, PAT4, Wildlife Computers, Redmond, USA) deployed on a variety of species (Figure S1)^2,3^. As the tag sensors are identical, the species tagged is irrelevant for building an error model for positioning purposes. Once detached from individuals, the tags are programmed to begin to transmit data via the ARGOS system (https://argos-system.cls.fr). These transmissions allow for a relatively accurate estimate of location to be determined and provide an associated measurement of SST. Thermistors used on PSATs are the same as those used on archival tags (M. Holland, Wildlife Computers, pers. comm.) so provides similar resolution and accuracy in temperature measurements.


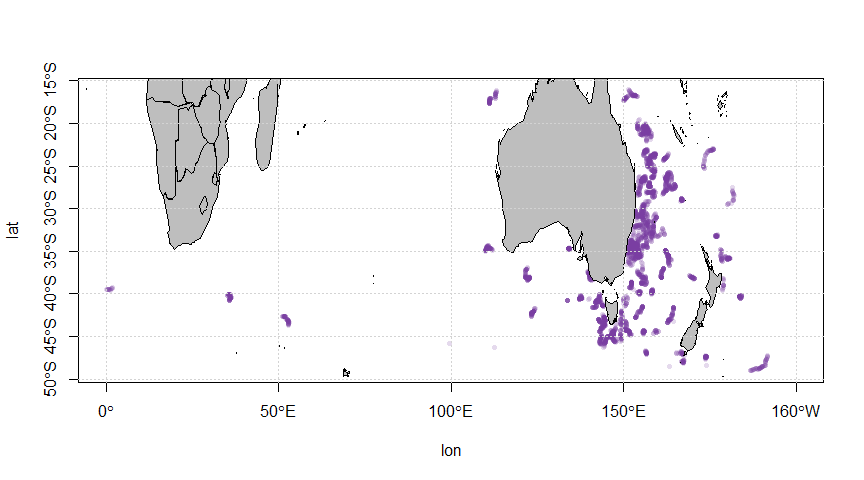


Figure S1. Locations of drifting pop-up satellite archival tags from which SSTs were used to calibrate the error model.

Using an optimally interpolated daily SST product, and the tag derived SST, a calibration data set with two measures of SST was generated. A linear model incorporating tag estimated SST ($SST_{tag}$ ) and remotely sensed SST ($SST_{satellite}$ ) was then used to assess bias and examine the distribution of errors.

There are two major sources of error and bias from tags deployed on marine animals (such as archival tags on SBT) relative to the remote sensing observations. Due to the fact that fish are not always absolutely at the ocean surface a single value of SST for a particular period in time (e.g. the period of geolocation estimation) must be calculated as a summary value across raw sensor readings from a range of ‘surface’ depths, which may or may not include those directly at the surface of the ocean. As a result, observations of SST from a tag may not represent true observations of surface temperature.

In contrast to the archival tags deployed on juvenile SBT, drifting PSATs probably do record an actual surface value given that they float in the very top layers. Therefore the following assumptions are applied; we allow the possibility of a constant bias but we assume the spread of errors from the quantity $\Delta T=SST_{tag}-SST_{satellite}$ are representive of the typical variability resulting from a diving SBT. The distribution of residual errors from the mean is of obviously important for spatial filtering results as it influences the likely locations.

Three SST error models were considered:

1. The first was a simple Gaussian, calculated directly from the mean and standard deviations of $\Delta T$.
2. To deal with outliers in the SST observations we modelled the distribution as a 2-component mixture of Normal and t-distributions. The negative log-likelihood is therefore:

$$\ln\{\mathcal{L}_{SST}(\Delta T;\mu,\sigma^{2},\nu)\}=-\sum_{i=1}^{n} \ln(p N(\Delta T_{i,}\mu,\sigma^{2})+(1-p)f(\Delta T_{i},\nu_{SST}))$$

where $f(.,\nu)$ is the student's t-distribution PDF, where $\nu$ is the degrees of freedom (df). The parameters $\mu, \sigma^{2}, p, \nu$ were estimated by numerical minimization.

1. A variant of the model above (2) where $\mu, \sigma^{2}$ were set to 0 and 1, respectively and $\nu,p$ were estimated.

A strong linear relationship between $SST_{tag}$ and $SST_{satellite}$ was apparent in the raw data (Figure S2). However, the residuals of the simple Gaussian model (model 1) showed departures from normality (Figure S3). The error distribution showed the presence of heavy tails in the residuals and that the distribution peaked around $\Delta T=0$. The mean $\Delta T$ value was 0.14^o^C (Table S1).

The mixture model (model 2) estimated a mean $\Delta T$ value of 0.16^o^C (Table S1). Based on a mixing probability of p=0.81 and the AIC score, this model was considered to represent the most likely distribution for $\Delta T$ observations. The variant on the mixture model (model 3) performed poorly, with a higher AIC score, and was not considered further.

Table S1. Parameter estimates for $\Delta T$ from the three candidate error models.

| Error model | Parameter | Value | AIC |
| --- | --- | --- | --- |
| Gaussian | mean | 0.14 | 8753.2 |
|  | SD | 0.72 |  |
| Mixture | p | 0.81 | 8020.5 |
|  | df | 6.83 |  |
|  | mean | 0.16 |  |
|  | SD | 0.52 |  |
| Variant mixture | p | 0.99 | 9359 |
|  | df | 1.18 |  |


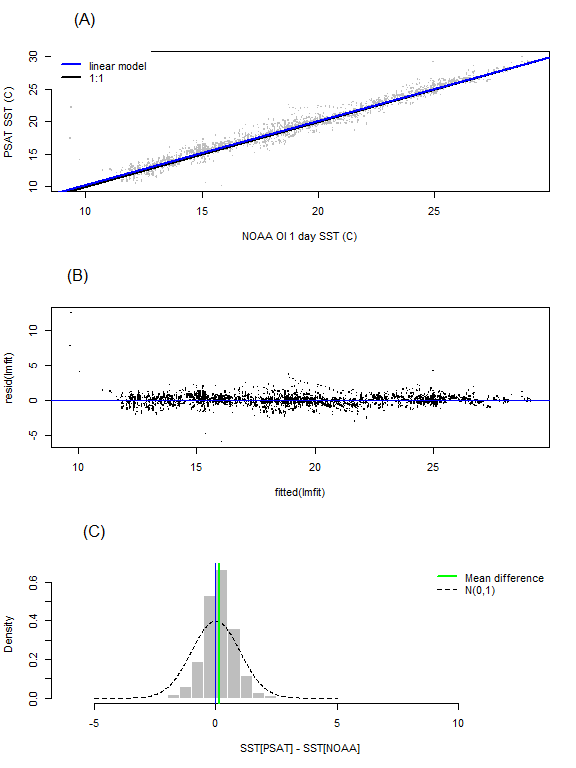


Figure S2. (A) Relationship between SST[tag] and satellite SST (SST[NOAA]). (B) Fitted vs predicted values from the linear model in (A). (C) Distribution of the difference between PSAT and remote sensing (SST[NOAA]) SST measurements.


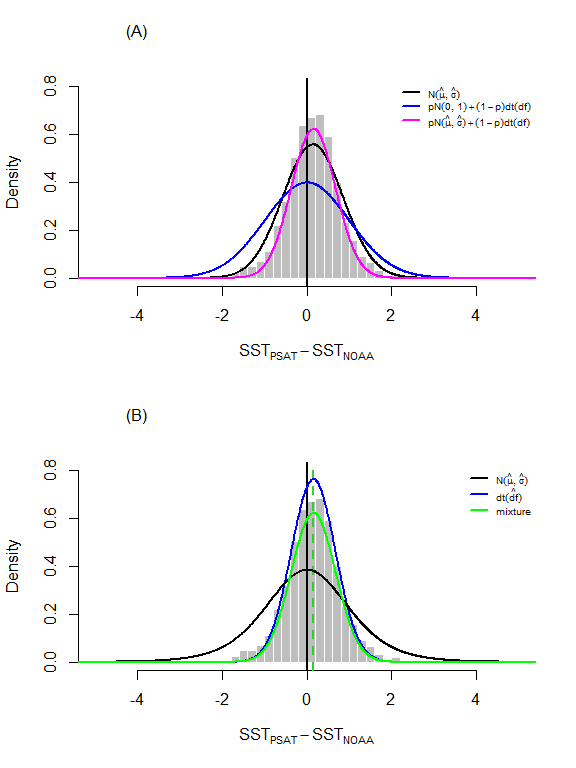


Figure S3. Comparison of the three candidate error distribution models. Gaussian error distribution (black line); mixture of Gaussian with estimated mean and variance and t-distribution model (magenta line) and mixture of N(0,1) and t-distribution (blue line).

In positioning the SBT tracks used in this study an estimate of SST was extracted using averaged ambient water temperature observations from the top 20m of the water column. Initial data exploration showed that the top 20m of the water column was highly mixed and there was little difference in SSTs calculated across these depths and those calculated across shallower depth ranges. The selected error model in Table S1 was applied to these observed SST values from the tags against optimally interpolated SST data in the state space model described above.

### 1.2.2 Maximum depth and bathymetry data

Bathymetric data in relation to fish diving records can be highly informative in determining position; if a fish dives to a depth much deeper than the bathymetry at that position it is unlikely to have visited that location. Here the TerrainBase^4^ global bathymetry data set was used Let $D_{i}$ be the bathymetric depth value for a grid cell and $D_{max}$ be the deepest depth record from each tag over a given observation period (e.g. the time period being used for geolocation). The data-likelihood of given region is calculated using the following formulae:

$$\begin{matrix} h_{i}(\Delta D_{i}) & = & \beta_{1}(D_{i}-D_{max})+\beta_{0} \\ \mathcal{L}_{depth}(D_{i}, D_{max}, \beta_{0}, \beta_{1}) & = & h_{i}^{-1}(D_{i}) \\ & = & \frac{\exp[h(D_{i})]}{1+\exp[h(D_{i}]} \end{matrix}$$

This is a logistic relationship with depth and a binomial log-likelihood. We have no accurate information on the value of $\beta_{0}$ and $\beta_{1}$ so in some sense, these can be regarded as dictating an informative prior on the probability of location given an observation of $D_{max}$ and a gridded bathymetry product with unknown levels of imprecision. The intent of employing this approach is that we assume values of $D_{max}\ll D_{i}$ should not be very informative about the location. Values where $D_{max}\approx D_{i}$ may be somewhat informative as uncertainty in location as well as uncertainty in the true bathymetric value may mean that $D_{max}$ could be greater than $D_{i}$. However if $D_{max}\gg D_{i}$ for a given location then we expect that this point is highly unlikely as a candidate location in the filter. In other words, some error could be tolerated but the maximum depth should not greatly exceed the bathymetry data.

**1.3 Estimation of most probable tracks**

The process model was combined with an error model in a standard Hidden Markov model (HMM) framework where the likelihood is calculated in the manner described in Pedersen et al.^5^. The initial state (location) was known from the GPS location taken at the time of tag releases. Hence the initial state vector $\mathbf{X}_{0}$ consists of a 1 for the cell containing the known release location and is 0 elsewhere. The most likely position at any given time was taken as the pair made up of the weighted average of X or Y coordinates in the grid where the weights were taken as the probability of each grid location in the HMM.

1. Seasonal cycles in ocean temperature and productivity and SBT migration

Monthly surface chlorophyll-a (Chl-a) and SST values derived from remote sensing across the range of juvenile SBT were investigated for regional differences in productivity and thermal characteristics. Surface Chl-a data was extracted from the MODIS 4km monthly Chl-a concentration global data set covering the period July 2002 to December 2015^1^ and SST was derived from the monthly reconstructed Reynolds SST covering the period February 2000 to January 2016^2^. Mean SST and the 90th percentile of Chl-a values ($P_{90})$ for each month were calculated for each of the six regions. The 90th percentile of Chl-a was calculated rather than the mean for two reasons. First, preliminary analysis suggested that some Chl-a values were anomalously high and likely to be erroneous. Second, large pulses of primary production, rather than high average production sustained through time, may be more likely to mediate increased flow of energy to higher trophic levels. Accordingly, these events are more likely to be captured by a calculation related to seasonal maxima, rather than averages which tend to remain more constant.


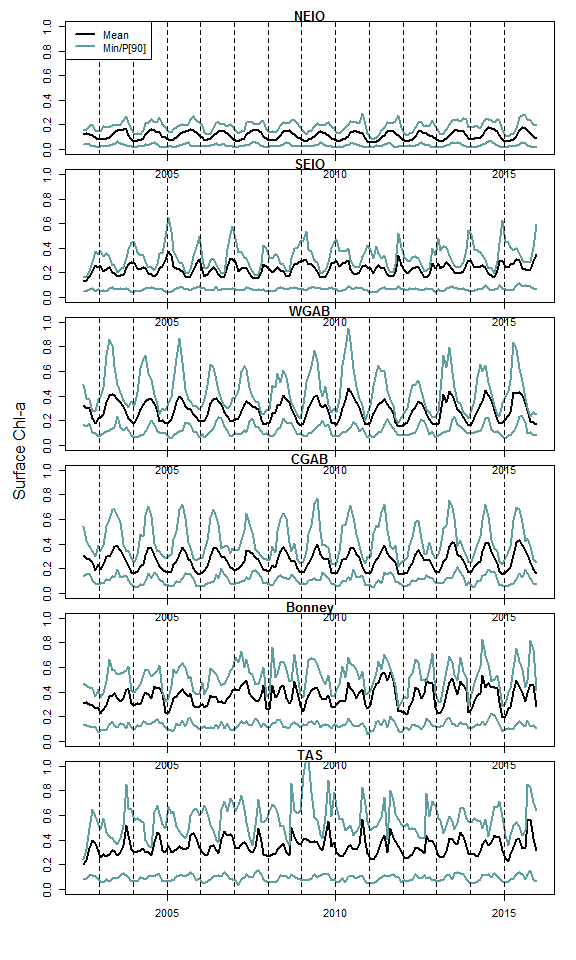


Figure S4. Mean, minimum and 90th percentile (P_90_) values of surface Chl-a in each of the six areas of residence by juvenile southern bluefin tuna.

1. **Changes in migration extent with age**

The data showed a clear trend of migration extent increasing over ages 1-6 years. Noting that the number of fish in the age 1 and age 5+ classes are small (Table S2), the maximum extent of movements to the west and south increased with age (Figure S5). Age 1 fish appear to be largely restricted to the coastal and shelf waters but age 2 fish made migrations west to around 80°E and to waters south of Tasmania. Ages 3 and 4 distribution was largely similar although there is some evidence of progressively decreasing GAB residence. The small sample (N=4) of age 6 positions showed very little usage of the GAB area. There was little evidence of systematic relationship of return and departure timing to and from the GAB being related to age (Figure S6).


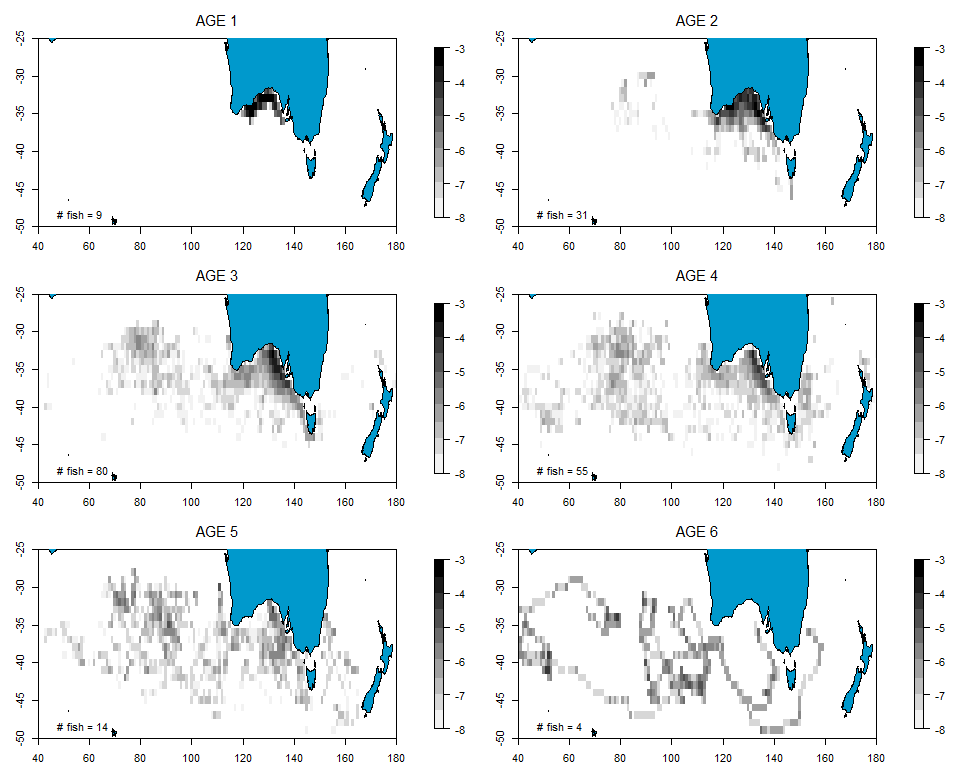


Figure S5. Log-density of SBT locations split by age class.

Table S2. Mean of maximum longitude and latitude positions by age class. A clear trend westward and southward is apparent with age. Note that the sample sizes for age 1 and age 5+ fish are small.

| Age | N | Longitude | Latitude |
| --- | --- | --- | --- |
| 1 | 9 | 122.7 | -35.2 |
| 2 | 31 | 118.7 | -37.9 |
| 3 | 80 | 106.1 | -40.6 |
| 4 | 55 | 90.5 | -43.2 |
| 5 | 14 | 84.6 | -45.6 |
| 6 | 4 | 54.40 | -46.00 |


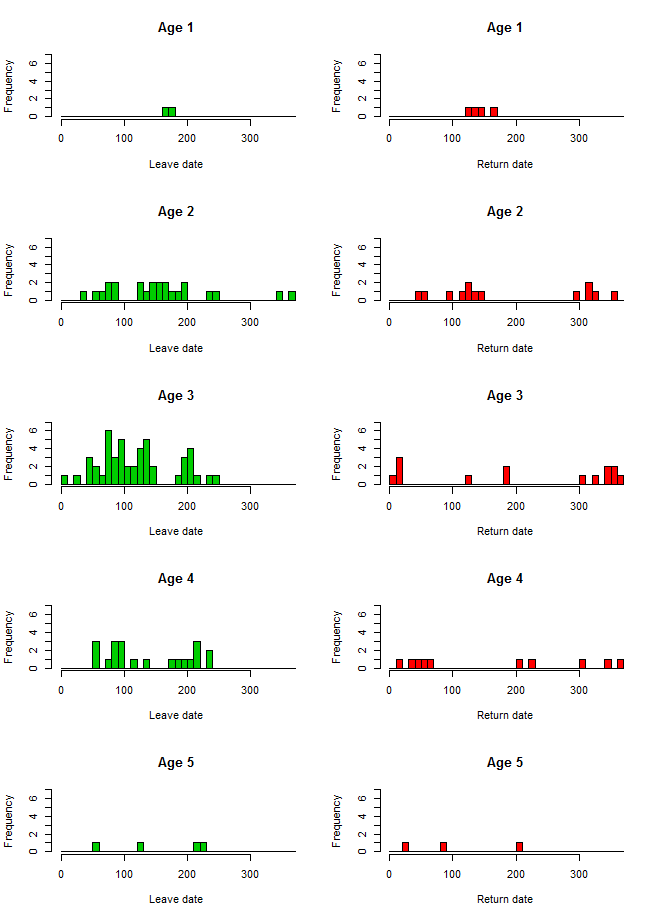


Figure S6. Frequency distribution of leave (left column) and return (right column) day of the year by age class.

**References**

1. Basson, M., Bravington, M. V., Hartog, J. R., & Patterson, T. A. (2016). Experimentally derived likelihoods for light‐based geolocation. Methods in Ecology and Evolution, 7(8), 980-989.
2. Patterson, T. A., Basson, M., Bravington, M. V., & Gunn, J. S. (2009). Classifying movement behaviour in relation to environmental conditions using hidden Markov models. Journal of Animal Ecology, 78(6), 1113-1123.Evans et al 2011
3. Banzon, V., Smith, T. M., Chin, T. M., Liu, C., and Hankins, W., 2016: A long-term record of blended satellite and in situ sea-surface temperature for climate monitoring, modeling and environmental studies. Earth Syst. Sci. Data, 8, 165–176, doi:10.5194/essd-8-165-2016
4. National Geophysical Data Center/NESDIS/NOAA/U.S. Department of Commerce. (1995) TerrainBase, Global 5 Arc-minute Ocean Depth and Land Elevation from the US National Geophysical Data Center (NGDC). Research Data Archive at the National Center for Atmospheric Research, Computational and Information Systems Laboratory. http://rda.ucar.edu/datasets/ds759.2/
5. Thygesen, U. H., Pedersen, M. W., & Madsen, H. (2009). Geolocating fish using hidden Markov models and data storage tags. In Tagging and tracking of marine animals with electronic devices (pp. 277-293). Springer, Dordrecht.
6. Hu, C., Z. Lee, and B.A. Franz (2012). Chlorophyll-a algorithms for oligotrophic oceans: A novel approach based on three-band reflectance difference, J. Geophys. Res., 117, C01011, doi:10.1029/2011JC007395.

1. [↑](#footnote-ref-1)
